# Supported progressive resistance exercise training to counter the adverse side effects of robot-assisted radical prostatectomy: a randomised controlled trial

**DOI:** 10.1007/s00520-021-06002-5

**Published:** 2021-01-23

**Authors:** Ruth E. Ashton, Jonathan J. Aning, Garry A. Tew, Wendy A Robson, John M Saxton

**Affiliations:** 1 Department of Sport, Outdoor and Exercise Science, University of Derby, Derby, UK; 2 Bristol Urological Institute, North Bristol NHS Trust, Southmead Hospital, Bristol, UK; 3 Department of Sport, Exercise & Rehabilitation, Faculty of Health & Life Sciences, University of Northumbria at Newcastle, Room 239, Northumberland Building, Newcastle-upon-Tyne, NE1 8ST UK; 4 Department of Urology, Freeman Hospital, Newcastle-upon-Tyne, UK

**Keywords:** Prostate cancer, Resistance exercise, Robot-assisted radical prostatectomy, Cardiometabolic

## Abstract

**Purpose:**

To investigate the effects of a supported home-based progressive resistance exercise training (RET) programme on indices of cardiovascular health, muscular strength and health-related quality of life (HR-QoL) in prostate cancer (PCa) patients after treatment with robot-assisted radical prostatectomy (RARP).

**Methods:**

This study was a single-site, two-arm randomised controlled trial, with 40 participants randomised to either the intervention or control group over a 10-month period. In addition to receiving usual care, the intervention group completed three weekly RET sessions using resistance bands for 6 months. Participants performed 3 sets of 12–15 repetitions for each exercise, targeting each major muscle group. The control group received usual care only. Brachial artery flow-mediated dilatation (FMD) was the primary outcome and assessed at baseline, 3 and 6 months. Secondary outcomes included body weight, body fat, aerobic fitness, strength and blood-borne biomarkers associated with cardiometabolic risk.

**Results:**

There was no significant difference between the groups in FMD at 3 or 6 months. However, there were improvements in aerobic exercise capacity (*P* < 0.01) and upper- (*P* < 0.01) and lower-limb (*P* = 0.01) strength in favour of the RET group at 6 months, accompanied by greater weight loss (*P* = 0.04) and a reduction in body fat (*P* = 0.02). Improvements in HRQoL were evident in the RET group at 3 and 6 months via the PCa-specific component of the FACT-P questionnaire (both *P* < 0.01). Five adverse events and one serious adverse event were reported throughout the trial duration.

**Conclusion:**

This study demonstrates that home-based RET is an effective and safe mode of exercise that elicits beneficial effects on aerobic exercise capacity, muscular strength and HR-QoL in men who have undergone RARP.

**Trial registration:**

ISRCTN10490647.

**Supplementary Information:**

The online version contains supplementary material available at 10.1007/s00520-021-06002-5.

## Introduction

Robot-assisted radical prostatectomy (RARP) is an established minimally invasive treatment for localised prostate cancer (PCa). Men undergoing RARP are well-counselled about the common side effects of surgery, including erectile dysfunction and urinary incontinence, which can have a negative impact on confidence, masculinity and health-related quality of life (HR-QoL) [4, 5, 34]. Men undergoing surgical treatment for PCa are often considered to be in good general health, perhaps due to younger age [26] and fewer comorbidities [44]. However, recent cross-sectional data suggest that men treated with RARP an average of 11.7 months previously have an approximate one in five chances of a cardiovascular-related event within the next 10 years and a similar risk of suffering from clinically important levels of fatigue [6]. This evidence of elevated cardiovascular risk is consistent with data from 100 consecutive men diagnosed with localised PCa which showed an intermediate to high Framingham risk score in >95% of the patients [17]. Muscular strength may also be adversely affected by surgery in this population. A recent small-scale study reported a reduction in upper- and lower-limb strength, which was accompanied by decreases in upper- and lower-limb lean body mass, following radical prostatectomy [43]. This evidence provides a rationale for developing strategies to improve indices of cardiovascular health and skeletal muscle function in men with localised PCa undergoing RARP.

Current guidance recommends that men with PCa participate in structured exercise programmes to improve their health and HR-QoL [13, 33]. There is no specific exercise guidance for men after treatment with RARP, other than recommendations for cancer patients experiencing urinary incontinence [25], and this reflects the current lack of evidence for this PCa sub-population [10]. However, a 6-month programme of aerobic (walking) exercise was shown to improve indices of cardiovascular health but not erectile dysfunction after surgical treatment for clinically localised PCa [27]. Furthermore, a 12-week programme of twice-weekly resistance exercise training (RET), which included pelvic floor exercise, improved physical function, continence rate and quality of life versus pelvic floor exercise alone after radical prostatectomy [37].

RET is considered to be safe, and in addition to increasing skeletal muscle mass, it has been shown to be effective for improving indices of cardiovascular health, symptoms of cancer-related fatigue and HR-QoL in men with PCa and older adults [7, 16, 34, 35]. The majority of studies examining the effects of RET in clinical populations and older adults have involved supervised exercise [7]. Because supervised exercise requires travel to an exercise facility, which can be costly and time-consuming for patients, it can act as a barrier to participation. However, a key advantage of RET is that it is a highly accessible form of exercise that patients can perform at home following a short instruction period. Home-based RET can be relatively low-cost and convenient, particularly for older individuals who may have less confidence to use leisure centres and gym facilities [45]. Hence, the main aim of this study was to investigate the effects of a home-based progressive RET programme (encompassing a short period of supervised instruction, followed by remote support) on indices of cardiovascular health in men with PCa undergoing RARP and, secondly, to assess the impact of the exercise programme on muscular strength and HR-QoL.

## Methods

### Trial design

This two-armed randomised controlled trial was designed with patient and public involvement. A panel consisting of 28 patients at different stages of treatment (recruited from PCa clinics and the local PCa support group) and healthcare professionals helped to formulate the study. Furthermore, a patient representative was appointed to the Trial Management Group and was consulted on all aspects of the study.

### Participants and setting

Potentially eligible patients attending outpatient clinics were approached by the urology team and informed about the study. A patient letter and information sheet were provided. Patients keen to participate were contacted by a member of the research team and screened to ensure they met the inclusion criteria of a PCa diagnosis, undergone RARP in the previous 8–12 weeks and completed a cardiopulmonary exercise test (CPET) in the previous 4 months. The study exclusion criteria were receiving any other cancer treatment, planned surgery within the next 3 months and participating in another clinical trial where concurrent participation was deemed inappropriate by a clinical investigator. Consenting men underwent checks of resting heart rate and blood pressure and cardiac function using a 12-lead echocardiogram (ECG). The ECG was conducted at rest and was checked by a medical professional. Participants with no contraindications to exercise were allowed to continue into the study.

All patients were recruited from the Freeman Hospital, Newcastle-upon Tyne. The study received ethical approval from the NHS Research Ethics Committee South Scotland and was prospectively registered on the International Standard Randomised Controlled Trial Number database (ref: ISRCTN10490647). All participants provided written informed consent before enrolment. The Consolidated Standards of Reporting Trials (CONSORT) guidelines were followed to guide the reporting of this study [41].

### Randomisation and allocation concealment

Participants were randomised to either RET or usual care, with both groups receiving standard medical treatment (including pre- and post-operative care). Participants were randomly assigned (1:1) using a computer-generated block randomisation schedule (http://www.randomization.com) with a block of size 4. The block size and allocation sequence were not disclosed to ensure concealment. On completion of screening and the baseline assessment, the lead researcher emailed the randomisation administrator who responded with group allocation (RET or usual care).

### Intervention

The intervention group completed three weekly sessions of RET using resistance bands for 6 months. Participants completed 3 sets of 12–15 repetitions for each exercise in a circuit [2]. All participants performed 8–10 exercises which targeted the major muscle group such as legs (squat, leg press, quick kicks), abdominals (trunk curl-up, lower abdominal crunch, side bend), back (bent-over row, reverse flies), chest (chest press), shoulders (upright row, lateral raise, front raise) and arms (bicep curl and either elbow extension or elbow kick back). Exercises were performed with 30–60-s interpolated rest intervals until 3 sets of each exercise had been performed. The exercise programme was progressed according to the American College of Sports Medicine (ACSM) guidelines and the OMNI-Resistance Exercise Scale (OMNI-RES) [39]. The OMNI-RES is a perceived exertion scale for resistance exercise that helps to control intensity during strength training exercises and can be applied to both men and women. Participants were instructed either to alter the hand/foot position on the band to increase resistance or to progress onto the next level of resistance band once 7–8 was reached on the OMNI-RES [15]. All participants began on the easiest of the resistance bands for a minimum of 1 week. This enabled all participants to understand the movements involved in each exercise with slow progression of the exercises to help avoid any injury.

#### Supervision and remote support for the intervention group

Table 1 describes the level of exercise supervision over the course of the trial. The aim of the supervised sessions was to ensure that each participant received individual face-to-face tuition on the appropriate exercise techniques in a designated room at the hospital. Thereafter, patients were remotely supported by weekly telephone calls lasting approximately 10 min until 3 months. Telephone calls were used to discuss any issues and challenges patients had faced with regard to adherence, exercises and aches and pains, along with advising on exercise progression. After the third month, participants received no further contact with the research team until the 6-month outcome assessment session. An exercise booklet provided instructions for all exercises included in the RET programme and was used to record weekly RET activity levels, including the exercises performed, session duration and frequency and OMNI-RES score, over the 6 months.

**Table 1:** Level of supervision received by the RET group

Week	Supervision
1	3 supervised exercise sessions
2	2 supervised sessions and 1 in the home environment (i.e. unsupervised)
3–4	1 supervised session and 2 completed at home
5–12	All exercise sessions were unsupervised, but participants in the intervention group received weekly telephone contact from a member of the research team [23]
13–24	All sessions were unsupervised with no contact from the research team

N.B. Those who did not take up the invite of supervised sessions were contacted weekly via telephone from week 1

### Usual care

The control group received usual care only, comprising an outpatient follow-up appointment every 3 months and advice regarding incontinence. They were instructed to continue with their usual activity levels, but no exercise guidance was provided. Usual care for both groups was not affected by this trial.

### Outcome measures

All outcomes were assessed at baseline (prior to randomisation) and at 3- and 6-month follow-up. In addition to the primary and secondary outcomes, participant demographic characteristics such as age, ethnicity, time since surgery, comorbidities and pre-operative prostate-specific antigen (PSA) level were recorded at baseline.

#### Primary outcome

Flow-mediated dilatation (FMD) was the primary outcome. FMD is predictive of future cardiovascular events [46], and there is evidence that men treated with RARP are at elevated risk [6, 17]. FMD assesses the ability of the brachial artery to dilate in response to shear stress to test endothelial function. Participants laid in the supine position for 5 min prior to the test. A manual sphygmomanometer was placed distal to the olecranon process, and a resting measurement of vessel diameter was performed for 1 min before cuff inflation to a pressure 50 mmHg above systolic blood pressure (SBP). Occlusion was maintained for 5 min. Recordings were restarted 30 s before cuff release and continued for a further 3 min thereafter [46]. FMD measurements were completed at each assessment visit. Measurements of the artery diameter were taken, along with measures of shear rate. Analysis of FMD recordings was undertaken using Cardiovascular Suite software (Quipu v3.4, 2018). Measurements of baseline diameter (mm), maximum diameter (mm), recovery diameter (mm), baseline shear rate (s^−1^), maximum shear rate (s^−1^), area to maximum and FMD (%) were all recorded.

#### Secondary outcome measures

Participants arrived fasted for venous blood sample collection which were analysed for blood lipid, insulin and glucose levels. Anthropometric variables, associated with cardiometabolic risk, were also assessed in the fasted state, including body mass index (BMI), waist circumference and skinfold analysis (triceps, subscapular, biceps, iliac crest, supra-spinal, abdominal, front thigh, medial calf). Exercises from the senior fitness test were used to evaluate upper- and lower-limb strength [40]. Lower-limb strength was assessed using the chair sit-to-stand test, and upper-limb strength was assessed using the bicep curl test, requiring men to repeatedly lift an 8 lb. (3.63 kg) weight for 30 s [40].

Submaximal aerobic capacity was assessed using the Bruce ramp protocol on a motorised treadmill (Life Fitness, Next Gen 9500 Treadmill, Cambridge, UK) [39]. The exercise test was terminated when the participant reached 15 on Borg’s 6-20 Rating of Perceived Exertion Scale [28] or for safety reasons if the participant demonstrated an abnormal response to exercise (e.g. very high heart rate, chest pain, light headedness). V̇O_2_ peak was estimated from the level reached by the participant and using the ACSM Guidelines for Exercise Testing and Prescription [39].

Questionnaires were used to assess QoL (EQ-5D-5L [48] and FACT-P [19]), fatigue (Brief Fatigue Inventory [31]) and self-reported exercise behaviour (modified Godin Leisure Time Exercise Questionnaire [3]). All permissions were sought prior to questionnaire use.

### Blinding

Due to the nature of the intervention, participants were not blinded to group allocation. Assessments at 3 and 6 months that could be influenced by the researcher (e.g. aerobic capacity) were conducted by a trained research assistant blinded to group allocation to minimise the risk of bias. Analysis of blood samples and FMD data were also conducted blindly. Questionnaires were completed by participants independently and checked by a researcher for completeness.

### Sample size estimation

The trial was powered to detect a 2.2% absolute difference between groups in FMD at 3 months. Previous studies indicate that this is a clinically meaningful difference in terms of future cardiometabolic risk [8, 51] and one that is realistic to expect in a parallel-group study in which one group receive a 3-month structured exercise programme [22]. Assuming a standard deviation of 2.8% [21], the anticipated effect size was approximately 0.80. The power calculation indicated that to observe a difference of this magnitude with 80% power and 5% two-sided significance, a total sample size of 52 participants was required. Allowing for 15% attrition, we aimed to recruit 60 participants to the trial, with 30 participants randomised to each group.

### Data analysis and missing data

Normality of distribution for the outcome measures was tested using the Shapiro Wilks test, and assumptions were tested prior to analysis. The effect of the intervention was evaluated using an analysis of covariance (ANCOVA) model, with the baseline value of the outcome included as the covariate. The treatment effect (intervention minus control) is presented with 95% confidence intervals (CI). Multiple imputation was used for missing data prior to an intention-to-treat analysis being conducted [30]. There were missing data for some variables, and further details are provided in the supplementary material. Analyses run on the dataset were pooled according to Rubin’s (1987) rules. All analyses were conducted using IBM SPSS Statistics Version 22 (IBM United Kingdom Limited, Hampshire, UK).

## Results

### Participants

A CONSORT diagram illustrating recruitment, randomisation and completion is presented in the supplementary material. Of 73 eligible participants, 42 men were recruited to the trial and randomised over a 10-month period from June 2017 to April 2018. Participant demographic information is presented in Table 2.

**Table 2:** Participant demographics at baseline

	Exercise group (*n* = 20)	Usual care (*n* = 22)
Age (years)	64.6 ± 6.2	66.9 ± 6.8
Stature (cm)	176.9 ± 7.8	175.8 ± 6.5
Body mass (kg)	88.0 ± 13.3	87.6 ± 13.9
Body mass index (kg/m^2^)	28.1 ± 3.5	28.3 ± 4.1
White British *n* (%)	19 (95%)	22 (100%)
Weeks since surgery	10 ± 1	11 ± 2
Pre-operative PSA (ng/ml)	12.1 ± 10.9	11.9 ± 11.3
Hypertension *n* (%)	14 (33.3%)	8 (19.0%)
Hypercholesterolemia *n* (%)	6 (14.3%)	9 (21.4%)
T2DM *n* (%)	4 (9.5%)	1 (2.4%)
Asthma *n* (%)	4 (9.5%)	0 (0%)
Atrial fibrillation *n* (%)	2 (4.8%)	0 (0%)
Arthritis *n* (%)	0 (0%)	2 (4.8%)
Depression *n* (%)	0 (0%)	2 (4.8%)

Data are presented as mean ± standard deviation unless stated otherwise. *PSA* prostate-specific antigen, *T2DM* type 2 diabetes mellitus

### Intervention adherence

The RET group completed 8–10 exercises per session, with each session lasting 20–30 min. Adherence to the RET intervention in the first 3 months was 94.1% ± 10.5% with a mean of 1.9 ± 3.8 sessions missed. During the first 3 months, one participant suffered a pulmonary embolism judged to be unrelated to the study, and this patient was unable to complete 2 weeks of the programme as a consequence. In the first 3 months, the three main reasons for missing RET sessions were a bad back, common cold and holidays. In months 3–6, adherence in the RET group decreased slightly to 77.7% ± 29.8%, with an average of 8.0 ± 10.6 sessions missed. The three main reasons for missing sessions in months 3–6 included becoming a father, Christmas celebrations, a rotator cuff injury and holidays, and three participants did not return their diaries for analysis.

### Primary and secondary outcomes

Baseline data are presented in Tables 3 and 4. There was no significant difference between the groups for FMD variables at 3 or 6 months (Table 5). However, the RET group experienced improvements in many of the secondary outcomes at the 3- and 6-month follow-ups (Table 5). The adjusted mean difference in submaximal aerobic exercise stage, time and estimated V̇O_2_ peak scores were 7.3 (3.0, 11.6), *P* < 0.01, 142 s (55, 232), *P* < 0.01 and 7.8 ml/kg/min (3.2, 12.5), *P* < 0.01 at 3 months respectively and 6.8 (2.4, 11.1), *P* < 0.01, 140 s (54, 226), *P* < 0.01 and 8.5 ml/kg/min (3.8, 13.1), *P* < 0.01 at 6 months, respectively, in favour of the RET group. The adjusted mean upper and lower body strength scores were 3.6 (1.7, 5.5) reps, *P* < 0.01 and 3.1 (1.0, 5.2) reps, *P* < 0.01 at 3 months, respectively, and 4.3 (1.2, 7.3) reps, *P* < 0.01 and 3.2 (0.6, 5.9) reps, *P* = 0.02 at 6 months, respectively, again, in favour of the RET group. In addition, the adjusted mean difference in body mass, percentage fat and sum of 8 skinfolds were − 3.1 kg (−6.0, −0.2), *P* = 0.04, −1.9% (−3.5, −0.4), *P* = 0.02 and − 13 mm (−25, −1), *P* = 0.03 at 6 months, respectively, in favour of the RET group. However, there were no significant differences between the groups in resting blood pressure, resting heart rate or blood biomarkers.

**Table 3:** Baseline physiological measures

Outcome	Exercise group	Usual care
	Mean ± SD	Mean ± SD
Flow-mediated dilatation		
Baseline diameter (mm)	4.8 ± 0.6	4.8 ± 0.5
Max diameter (mm)	5.2 ± 0.9	5.1 ± 0.8
Recovery diameter (mm)	5.0 ± 0.6	5.1 ± 0.4
FMD (%)	6.9 ± 2.4	7.6 ± 2.4
Shear baseline (s^−1^)	76.8 ± 24.5	80.9 ± 20.8
Shear max (s^−1^)	390.8 ± 76.0	389.8 ± 143.3
Shear area to max	8558.9 ± 1638.5	8883.6 ± 4413.4
Cardiovascular health		
Resting heart rate (bpm)	71.7 ± 12.5	66.8 ± 9.3
SBP (mmHg)	138 ± 17	136 ± 15
DBP (mmHg)	82 ± 13	82 ± 9
QRisk-2 Score (%)	17.0 ± 7.7	18.0 ± 6.8
Anthropometric profile		
Body mass (kg)	88.0 ± 13.3	87.6 ± 13.9
Body mass index (kg/m^2^)	28.1 ± 3.5	28.3 ± 4.1
Waist circumference (cm)	99.6 ± 10.0	103.0 ± 9.7
Waist/hip	1.0 ± 0.1	1.0 ± 0.1
Fat (%)	18.3 ± 4.0	17.9 ± 4.6
Sum of 6 skinfolds (mm)	104 ± 25	101 ± 27
Sum of 8 skinfolds (mm)	136 ± 29	133 ± 35
Blood biomarkers		
Glucose (mmol/L)	6.2 ± 1.9	5.4 ± 0.6
Insulin (μU/ml)	34.2 ± 14.7	38.4 ± 21.5
HOMA-IR	2.3 ± 5.4	0.6 ± 0.4
Total Chol (mmol/L)	5.0 ± 1.1	5.0 ± 1.5
HDL (mmol/L)	1.3 ± 0.3	1.3 ± 0.3
LDL (mmol/L)	3.2 ± 0.8	2.8 ± 1.2
Non-HDL (mmol/L)	3.7 ± 1.0	3.8 ± 1.5
Triglycerides (mmol/L)	1.5 ± 0.7	1.4 ± 0.6
Total/HDL (mmol/L)	4.0 ± 0.9	4.0 ± 1.1
Submaximal aerobic exercise		
Stage	30.0 ± 11.4	29.8 ± 10.3
Time (secs)	580 ± 229	576 ± 205
Estimated VO_2_Peak (ml/kg/min)	38.6 ± 13.8	38.7 ± 12.5
Max heart rate (bpm)	143.2 ± 17.3	132.9 ± 17.3
Strength		
30-s bicep curl test (reps)	20.0 ± 5.7	19.4 ± 4.2
30-s chair stand test (reps)	17.9 ± 5.1	16.1 ± 5.3

*SD* standard deviation, *FMD* flow-mediated dilatation, *SBP* systolic blood pressure, *DBP* diastolic blood pressure, *HOMA-IR* insulin resistance, *Chol* cholesterol, *HDL* high-density lipoprotein cholesterol, *LDL* low-density lipoprotein cholesterol, *VO*_*2*_*Peak* maximal aerobic capacity, *reps* repetitions, *secs* seconds

**Table 4:** Baseline questionnaire measures

Outcome	Exercise group	Usual care
	Mean ± SD	Mean ± SD
Quality of life		
EQ-5D Index Score	0.9 ± 0.1	0.8 ± 0.2
ED-5D VAS	82 ± 13	78 ± 14
FACT-P		
Physical WB	25.4 ± 1.8	24.3 ± 2.9
Social WB	23.4 ± 3.5	22.5 ± 3.6
Emotional WB	21.6 ± 2.4	20.8 ± 3.7
Functional WB	21.5 ± 5.2	21.2 ± 5.5
Prostate cancer specific	34.5 ± 3.2	33.3 ± 5.2
FACT-P TOI	81.4 ± 8.7	78.8 ± 11.5
FACT-P Total Score	126.4 ± 12.1	122.2 ± 16.2
FACT-G Total Score	91.9 ± 10.0	88.8 ± 12.1
Fatigue		
BFI	1.2 ± 1.2	1.9 ± 1.4
Godin Leisure Time Exercise Questionnaire (modified)		
VA frequency (days/week)	0.0 ± 0.0	0.4 ± 1.5
VA duration (mins/session)	0.0 ± 0.0	1.1 ± 3.8
MIA frequency (days/week)	1.8 ± 2.8	1.1 ± 2.0
MIA duration (mins/session)	15.9 ± 27.6	25.7 ± 56.5
LIA frequency (days/week)	4.6 ± 2.2	5.3 ± 2.6
LIA duration (mins/session)	40.0 ± 31.5	59.6 ± 61.4
RET frequency (days/week)	0.4 ± 1.6	0.5 ± 1.6
RET duration (mins/session)	0.5 ± 2.2	2.5 ± 9.7

*SD* standard deviation, *VAS* visual analogue scale, *WB* well-being, *TOI* trial outcome index, *VA* vigorous aerobic activity, *MIA* moderate intensity aerobic activity, *LIA* low-intensity aerobic activity, *RET* resistance exercise training

**Table 5:** Cardiometabolic outcome measures

Outcome	3 Months		6 Months	
	Adjusted mean diff. between groups (95% CI)	*P* Value	Adjusted mean diff. between groups (95% CI)	*P* Value
Flow-mediated dilatation				
Baseline diameter (mm)	−0.1 (−0.4, 0.3)	0.4	−0.1 (−0.4, 0.2)	0.4
Max diameter (mm)	−0.04 (−0.4, 0.3)	0.7	−0.3 (−0.7, 0.1)	0.2
Recovery diameter (mm)	0.3 (−0.1, 0.7)	0.3	−0.4 (−0.9, 0.1)	0.09
FMD (%)	0.2 (−0.8, 1.2)	0.7	−1.6 (−4.7, 1.4)	0.2
Shear baseline (s^−1^)	12.5 (−27.9, 53.0)	0.5	−8.2 (−61.8, 45.4)	0.7
Shear max (s^−1^)	17.8 (−30.1, 65.7)	0.3	−12.2 (−77.0, 52.5)	0.3
Shear area to max	−1874.3 (−412.9, 5161.5)	0.5	−673.1 (−3741.5, 2395.3)	0.9
Cardiovascular health				
Resting heart rate (bpm)	10.9 (−5, 6)	0.7	1 (−6, 7)	0.8
SBP (mmHg)	−6 (−12, 1)	0.07	−7 (−15, 2)	0.1
DBP (mmHg)	−1 (−5, 3)	0.6	1 (−5, 7)	0.7
QRisk-2 Score (%)	0.1 (−2.7, 2.9)	0.9	0.3 (−2.7, 3.2)	0.8
Anthropometric profile				
Body mass (kg)	−1.4 (−3.6, 0.6)	0.2	−3.1 (−6.0, −0.2)	*0.04*
Body mass index (kg/m^2^)	−0.4 (−1.1, 0.2)	0.2	−1.0 (−1.9, −0.1)	*0.03*
Waist circumference (cm)	−1.3 (−3.9, 1.3)	0.3	−2.3 (−5.1, 0.5)	0.1
Waist/Hip	0.0 (0.0, 0.0)	0.6	−0.03 (−0.1, 0.006)	0.09
Fat (%)	−13.2 (−26.8, 0.4)	*0.01*	−1.9 (−3.5, −0.4)	*0.02*
Sum of 6 skinfolds (mm)	−2 (−3, 0)	*0.05*	−8 (−17, 1)	0.07
Sum of 8 skinfolds (mm)	−12 (−26, 1)	0.07	−13 (−25, −1)	*0.03*
Blood biomarkers				
Glucose (mmol/L)	0.03 (−1.0, 1.0)	0.9	−0.3 (−1.1, 0.4)	0.4
Insulin (μU/ml)	−15.2 (−46.2, 15.9)	0.3	−10.7 (−46.7, 25.2)	0.5
HOMA-IR	−0.3 (−1.0, 0.3)	0.3	−0.3 (−0.9, 0.3)	0.3
Total Chol (mmol/L)	−0.1 (−0.6, 0.4)	0.6	−0.4 (−0.9, 0.03)	0.07
HDL (mmol/L)	−0.04 (−0.5, 0.4)	0.6	0.01 (−0.1, 0.1)	0.08
LDL (mmol/L)	−0.2 (−0.8, 0.4)	0.8	−0.3 (−0.7, 0.1)	0.2
Non-HDL (mmol/L)	−0.2 (−0.5, 0.003)	0.5	−0.3 (−0.9, 0.2)	0.2
Triglycerides (mmol/L)	0.1 (−0.6, 0.8)	0.7	−0.2 (−0.6, 0.2)	0.4
Total/HDL (mmol/L)	0.01 (−0.5, 0.6)	0.9	−0.2 (−0.7, 0.3)	0.4
Submaximal aerobic exercise				
Stage	7.3 (3.0, 11.6)	*< 0.01*	6.8 (2.4, 11.1)	*< 0.01*
Time (secs)	142 (55, 232)	*< 0.01*	140 (54, 226)	*< 0.01*
Estimated VO_2_Peak (ml/kg/min)	7.8 (3.2, 12.5)	*< 0.01*	8.5 (3.8, 13.1)	*< 0.01*
Max heart rate (bpm)	3.1 (−2.8, 9.1)	0.2	6.8 (−0.8, 14.4)	0.07
Strength				
30-s bicep curl test (reps)	3.6 (1.7, 5.5)	*< 0.01*	4.3 (1.2, 7.3)	*< 0.01*
30-s chair stand test (reps)	3.1 (1.0, 5.2)	*< 0.01*	3.2 (0.6, 5.9)	*0.01*

*CI* confidence interval, *FMD* flow-mediated dilatation, *SBP* systolic blood pressure, *DBP* diastolic blood pressure, *HOMA-IR* insulin resistance, *Chol* cholesterol, *HDL* high-density lipoprotein cholesterol, *LDL* low-density lipoprotein cholesterol, *VO*_*2*_*Peak* maximal aerobic capacity, *reps* repetitions, *secs* seconds. Statistical significance: *P*<0.05

There were no changes in EQ-5D or BFI scores at 3 or 6 months (Table 6). However, there were significant changes in some aspects of the FACT-P. The adjusted mean difference in functional well-being, PCa specific and FACT-P Trial Outcome Index (TOI) were 1.9 (0.3, 3.5), *P* = 0.02, 2.3 (1.0, 3.7), *P* < 0.01 and 5.0 (1.9, 9.1), *P* < 0.01 at 3 months, respectively, and 1.9 (0.01, 3.8), *P* = 0.04, 3.1 (1.3, 5.0), *P* < 0.01 and 5.0 (1.8, 8.3), *P* < 0.01 at 6 months, respectively, in favour of the RET group. Significant changes were also evident for moderate-intensity activity at 3 months (22.1 [4.1, 40.0], *P* = 0.02).

**Table 6:** Questionnaire-based outcome measures

Outcome	3 Months		6 Months	
	Adjusted mean diff. between groups (95% CI)	*P* Value	Adjusted mean diff. between groups (95% CI)	*P* Value
Quality of life				
EQ-5D Index Score	0.02 (−0.04, 0.1)	0.6	0.1 (−0.01, 0.1)	0.1
ED-5D VAS	1 (−4, 7)	0.7	−1 (−6, 4)	0.7
FACT-P				
Physical WB	0.8 (−0.4, 2.0)	0.2	0.2 (−0.9, 1.3)	0.8
Social WB	1.2 (−0.4, 2.8)	0.1	−2.4 (−8.3, 3.5)	0.4
Emotional WB	−0.7 (−1.9, 0.6)	0.3	1.1 (−0.1, 2.3)	0.08
Functional WB	1.9 (0.3, 3.5)	*0.02*	1.9 (0.01, 3.8)	*0.04*
Prostate cancer specific	2.3 (1.0, 3.7)	*<0.01*	3.1 (1.3, 5.0)	*<0.01*
FACT-P TOI	5.0 (1.9, 9.1)	*<0.01*	5.0 (1.8, 8.3)	*<0.01*
FACT-P Total Score	5.3 (0.7, 9.8)	*0.03*	4.2 (−1.6, 10.1)	0.2
FACT-G Total Score	3.1 (−0.8, 7.0)	0.1	0.9 (−4.0, 5.7)	0.7
Fatigue				
BFI	−0.5 (−1.3, 0.3)	0.3	−0.1 (−0.9, 0.6)	0.7
Godin Leisure Time Exercise Questionnaire (modified)				
VA frequency (days/week)	0.1 (−0.4, 0.7)	0.7	0.4 (−0.1, 0.9)	0.2
VA duration (mins/session)	−27.2 (−66.3, 11.9)	0.2	−3.2 (−14.2, 7.8)	0.6
MIA frequency (days/week)	0.2 (−1.2, 1.6)	0.8	0.3 (−0.9, 1.5)	0.6
MIA duration (mins/session)	22.1 (4.1, 40.0)	*0.02*	16.9 (−8.1, 42.0)	0.2
LIA frequency (days/week)	−1.0 (−2.4, 0.4)	0.2	−0.7 (−2.2, 0.8)	0.4
LIA duration (mins/session)	−10.4 (−33.9, 13.2)	0.4	3.4 (−24.1, 30.9)	0.8
RET frequency (days/week)	2.6 (1.8, 3.5)	*<0.01*	1.9 (1.0, 2.9)	*<0.01*
RET duration (mins/session)	27.8 (20.7, 35.0)	*<0.01*	17.4 (10.8, 24.0)	*<0.01*

*CI* confidence interval, *VAS* visual analogue scale, *WB* well-being, *TOI* trial outcome index, *VA* vigorous aerobic activity, *MIA* moderate intensity aerobic activity, *LIA* low-intensity aerobic activity, *RET* resistance exercise training. Statistical significance: *P*<0.05

### Missing data

Approximately 38% of participants had some missing data over the 6 months of follow-up for FMD. All participants who attended follow-up sessions had blood taken; however, approximately 15% of participants did not have some sample results returned from the laboratory or the results were incomplete. The main reason for missing data points was participant drop-out.

### Adverse events

Five adverse events (hernia, accident resulting in back pain, abdominal pain, increased fasted insulin level, rotator cuff injury) and one serious adverse event (pulmonary embolism) were reported throughout the trial duration. Only the report of a rotator cuff injury was related to the trial and was the result of a participant choosing to progress to a more difficult resistance band despite being advised otherwise.

## Discussion

This is the first study to explore the health benefits of a predominantly home-supported progressive RET programme with remote supervision in PCa patients who have recently undergone RARP. The study demonstrates that RET can be implemented safely 8 weeks after RARP and can lead to improvements in a range of important health outcomes. Furthermore, there was a high level of adherence to home-based RET throughout the whole 6 months of the programme.

The results provide some evidence of improved cardiovascular health in the group allocated to RET. FMD was used as a simple, non-invasive measure of arterial endothelial function, which has been used previously in studies of exercise training in populations with elevated cardiovascular disease risk, thereby providing clinically relevant data regarding objective risk of CVD [46]. We observed no change in FMD at either the 3-month or 6-month time point in the patients allocated to the RET group. However, it is likely that the study was underpowered to detect changes in FMD, as an a priori power calculation indicated that 52 participants were needed to observe statistically significant differences in FMD between the groups. Other studies have reported a change in FMD from 6.2–8.3% to 9.7–11.8% after RET programmes, although the participants were young (18–35 years old), pre-hypertensive and healthy young (18 years old) adults, respectively [9, 36]. FMD could be a useful non-invasive technique for detecting changes in arterial function throughout treatment in PCa patients at elevated risk of cardiovascular disease, but as our results were inconclusive, further research is warranted.

This study demonstrated that RET improved aerobic capacity in men post-RARP. This is an important finding given that aerobic exercise capacity is known to decline with increasing age and diagnosed hypertension. Reductions in V̇O_2_Peak can exacerbate underlying CV conditions, impair activities of daily living and increase the risk of depression and functional dependence [14, 47]. Submaximal exercise testing, as employed in this study, is a means of predicting V̇O_2_Peak without performing maximal exercise and may be associated with fewer risks for clinical populations. This type of testing could allow clinicians to monitor patient progress and be used as a tool to motivate men in continuing with an exercise programme.

There were favourable but non-significant reductions in SBP at the 3- and 6-month follow-up time-points in the RET group. Approximately 50% of myocardial infarctions and strokes in the UK are attributed to hypertension, and so interventions to reduce SBP amongst men treated for PCa could contribute to a reduction in future cardiovascular events [11]. Importantly, hypertension was shown to be significantly more prevalent in a cohort of 100 consecutive men diagnosed with localised PCa [17], and CVD has been identified as one of the leading causes of death in men following RARP [38, 42]. Our results suggest that RET could be used as a highly accessible, non-pharmacological method of reducing arterial blood pressure in men with localised PCa treated with RARP. The design of RET programme used in this study also enables implementation during the current COVID-19 pandemic. Home-based programmes with remote support (via telephone, video-conferencing, etc.) prevent the need for patients to travel to exercise venues and allow for social distancing measures to be heeded. However, further research would be needed to determine the effectiveness of the initial instruction sessions if carried out virtually.

Improvements were observed in both upper- and lower-limb muscular strength in the RET group compared with usual care. As the average age of those recruited in this study was >60 years, this suggests that home-based RET could help to prevent or ameliorate the age-related loss of skeletal muscle mass and function (i.e. myopenia, sarcopenia, etc.) [2]. Improvements in upper- and lower-limb strength have previously been reported in studies of PCa after programmes of RET [20, 50]. However, aside from these improvements in skeletal muscle strength and function, there were significant changes in all body composition variables in favour of the RET group over 6 months of follow-up, suggesting RET is beneficial for promoting fat loss in PCa patients undergoing RARP, and this is consistent with data from other populations [49]. The effects of RET on body fat stores are potentially attributable to an increase in total daily energy expenditure on exercising days and as the involved skeletal muscles adapt to training, as some studies have shown increases in basal metabolic rates and fat oxidation following RET [1, 29].

Finally, the results of this study suggest that RET can improve patient-reported functional well-being and HR-QoL. This is noteworthy, given that studies have recently reported mental health issues and reduce HR-QoL in the years immediately after PCa diagnosis [12]. The significant difference between the two groups observed in this study suggests that RET is more beneficial than usual care [18] and could therefore have clinical implications regarding the advice and recommendations clinicians make for patients at this stage of their recovery. Participants reported improved PCa specific side effects via the FACT-P questionnaire. Therefore, it can be concluded that RET did not exert any negative effects on incontinence or erectile dysfunction and that participants in the RET group reported a perceived improvements in these commonly reported side effects of RARP. As a result, men could be counselled by healthcare professionals to not be concerned about the effect RET may have on their functional status especially with the programme being conducted in the home environment. RET did not appear to benefit fatigue but did not worsen reported fatigue levels. The lack of change could be due to the low fatigue levels reported by participants at baseline, and the study was probably underpowered to detect any small, but potentially important, difference in the fatigue outcome. However, other studies have reported that exercise has reduced the levels of fatigue in other cancer populations [32].

### Limitations

This study has a number of important limitations. Firstly, the study was underpowered to detect changes in the primary outcome, though significant beneficial effects were evident for many of the secondary outcomes (e.g. body weight, body fat, aerobic fitness, strength, FACT-P). Secondly, although a widely adopted multiple imputation technique was used to account for missing data points [24], missing data contributes to a loss of statistical power and potential bias. Adding to this, some participants were not fully compliant, with some exercise diaries lost or not returned for analysis. Thirdly, it is possible that due to patients being aware of their participation in an exercise trial, they undertook more daily exercise (in addition to RET), and this may have accounted for the observed increase in moderate-intensity physical activity and improvement in aerobic exercise capacity in this group. Fourthly, diet was not accounted for and may have improved due to participation in the trial, potentially leading to favourable reductions in body/fat mass. Finally, it is unclear how the intervention specifically impacted common side effects of RARP, including erectile dysfunction and urinary incontinence. Future research should expand on these preliminary findings and explore the impact of RET on key health outcomes in a larger multi-centre randomised controlled trial.

## Conclusions

This study highlights that RET is a safe, effective and feasible mode of exercise that elicits cardiometabolic health benefits in men who have undergone RARP. RET is an accessible form of exercise for PCa patients recovering from RARP and, when provided with appropriate information, demonstrates good adherence to the programme and reports few trial-related adverse events. Therefore, clinicians should consider discussing the benefits of exercise, including RET, to patients in the weeks following RARP.

## Supplementary information

ESM 1 (DOCX 40 kb)

ESM 2 (DOCX 21 kb)

## Data Availability

The research data support our published claims and comply with field standards.
