# Supplementary figures and images for: Supported progressive resistance exercise training to counter the adverse side effects of robot-assisted radical prostatectomy: a randomised controlled trial
Source: Support Care Cancer. 2021 Jan 23;29(8):4595–605. doi: 10.1007/s00520-021-06002-5 (PMC7822752; doi:10.1007/s00520-021-06002-5)

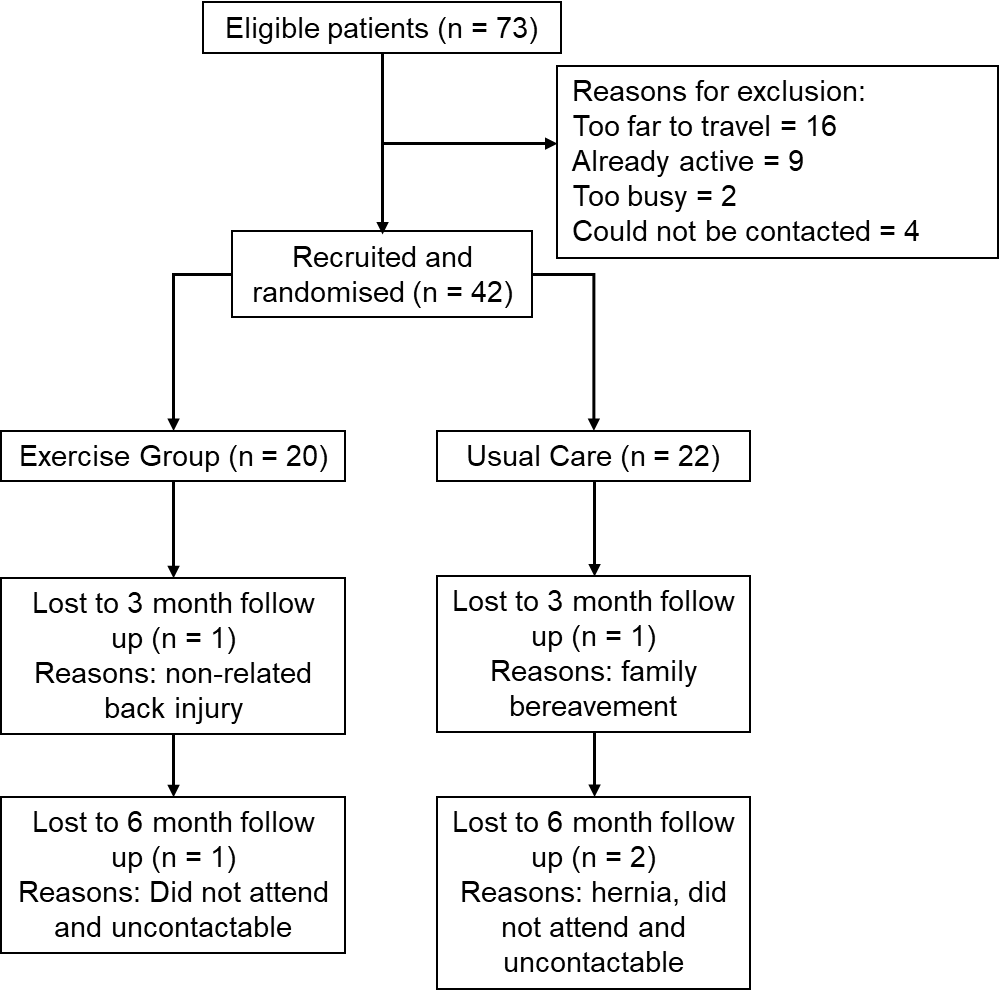


**Figure 1.** CONSORT flowchart of participant recruitment, randomisation, and completion.

Supplement: Supplementary file 1 — (DOCX 40 kb) [file 520_2021_6002_MOESM1_ESM.docx]
