# Supplementary material for: Supported progressive resistance exercise training to counter the adverse side effects of robot-assisted radical prostatectomy: a randomised controlled trial
Source: Support Care Cancer. 2021 Jan 23;29(8):4595–605. doi: 10.1007/s00520-021-06002-5 (PMC7822752; doi:10.1007/s00520-021-06002-5)
Supplement: Supplementary file 2 — (DOCX 21 kb) [file 520_2021_6002_MOESM2_ESM.docx]

**Table 1.** Missing data

| **Outcome** | **Baseline** | | **3 Month**  **Follow-up** | | **6 Month**  **Follow-up** | |
| --- | --- | --- | --- | --- | --- | --- |
|  | **RET** | **UC** | **RET** | **UC** | **RET** | **UC** |
| ***Flow Mediated Dilatation*** | | | | | | |
| Baseline Diameter (mm) | 6 (14.3%) | 11 (26.2%) | 12 (28.6%) | 13 (31.0%) | 10 (23.8%) | 13 (31.0%) |
| Max Diameter (mm) | 6 (14.3%) | 11 (26.2%) | 12 (28.6%) | 13 (31.0%) | 10 (23.8%) | 13 (31.0%) |
| Recovery Diameter (mm) | 6 (14.3%) | 12 (28.6%) | 12 (28.6%) | 13 (31.0%) | 10 (23.8%) | 13 (31.0%) |
| FMD (%) | 6 (14.3%) | 11 (26.2%) | 12 (28.6%) | 13 (31.0%) | 10 (23.8%) | 13 (31.0%) |
| FMDr (%) | 7 (16.7%) | 12 (28.6%) | 12 (28.6%) | 13 (31.0%) | 10 (23.8%) | 13 (31.0%) |
| Shear Baseline (s^-1^) | 9 (21.4%) | 11 (26.2%) | 12 (28.6%) | 13 (31.0%) | 10 (23.8%) | 13 (31.0%) |
| Shear Max (s^-1^) | 10 (23.8%) | 11 (26.2%) | 12 (28.6%) | 13 (31.0%) | 10 (23.8%) | 13 (31.0%) |
| Shear Area | 10 (23.8%) | 11 (26.2%) | 12 (28.6%) | 13 (31.0%) | 10 (23.8%) | 13 (31.0%) |
| Shear Area to Max | 10 (23.8%) | 12 (28.6%) | 12 (28.6%) | 15 (35.7%) | 10 (23.8%) | 13 (31.0%) |
| ***Blood Biomarkers*** | | | | | | |
| Glucose (mmol/L) | 3  (7.1%) | 1  (2.4%) | 4  (9.5%) | 7 (16.7%) | 3  (7.1%) | 4  (9.5%) |
| Insulin (μU/ml) | 6 (14.3%) | 2  (4.8%) | 4  (9.5%) | 9 (21.4%) | 5 (11.9%) | 8 (19.0%) |
| HOMA-IR | 6 (14.3%) | 2  (4.8%) | 6 (14.3%) | 9 (21.4%) | 6 (14.3%) | 8 (19.0%) |
| Total Chol (mmol/L) | 1  (2.4%) | 2  (4.8%) | 3  (7.1%) | 6 (14.3%) | 2  (4.8%) | 3  (7.1%) |
| HDL Cholesterol (mmol/L) | 1  (2.4%) | 2  (4.8%) | 3  (7.1%) | 6 (14.3%) | 2  (4.8%) | 3  (7.1%) |
| LDL Chiolesterol (mmol/L) | 6 (14.3%) | 7 (16.7%) | 7 (16.7%) | 8 (19.0%) | 7 (16.7%) | 7 (16.7%) |
| Non-HDL (mmol/L) | 4  (9.5%) | 4  (9.5%) | 3  (7.1%) | 6 (14.3%) | 2  (4.8%) | 3  (7.1%) |
| Triglycerides (mmol/L) | 1  (2.4%) | 2  (4.8%) | 3  (7.1%) | 6 (14.3%) | 3  (7.1%) | 3  (7.1%) |
| Total:HDL (mmol/L) | 1  (2.4%) | 2  (4.8%) | 3  (7.1%) | 6 (14.3%) | 2  (4.8%) | 3  (7.1%) |
| ***Cardiovascular Health*** | | | | | | |
| Resting Heart Rate (bpm) | 0  (0%) | 0  (0%) | 2  (4.8%) | 1  (2.4%) | 2  (4.8%) | 3  (7.1%) |
| Blood pressure (mmHg) | 0  (0%) | 0  (0%) | 2  (4.8%) | 1  (2.4%) | 2  (4.8%) | 3  (7.1%) |
| QRisk-2 Score (%) | 0  (0%) | 0  (0%) | 2  (4.8%) | 1  (2.4%) | 2  (4.8%) | 3  (7.1%) |
| ***Anthropometric Profile*** | | | | | | |
| Body Mass (kg) | 0  (0%) | 0  (0%) | 2  (4.8%) | 1  (2.4%) | 2  (4.8%) | 3  (7.1%) |
| BMI (kg/m^2^) | 0  (0%) | 0  (0%) | 2  (4.8%) | 1  (2.4%) | 2  (4.8%) | 3  (7.1%) |
| Waist Circumference (cm) | 0  (0%) | 3 (7.1%) | 2  (4.8%) | 2  (4.8%) | 2  (4.8%) | 3  (7.1%) |
| Waist:Hip | 0  (0%) | 3 (7.1%) | 2  (4.8%) | 2  (4.8%) | 2  (4.8%) | 3  (7.1%) |
| Skinfolds | 0  (0%) | 3 (7.1%) | 2  (4.8%) | 2  (4.8%) | 2  (4.8%) | 3  (7.1%) |
| ***Submaximal Aerobic Exercise*** | | | | | | |
| Stage | 0  (0%) | 0  (0%) | 2  (4.8%) | 1  (2.4%) | 4  (9.5%) | 3  (7.1%) |
| Time (secs) | 0  (0%) | 0  (0%) | 2  (4.8%) | 1  (2.4%) | 4  (9.5%) | 3  (7.1%) |
| Estimated V̇O_2_Peak (ml/kg/min) | 0  (0%) | 0  (0%) | 2  (4.8%) | 1  (2.4%) | 4  (9.5%) | 3  (7.1%) |
| Estimated METs | 0  (0%) | 0  (0%) | 2  (4.8%) | 1  (2.4%) | 4  (9.5%) | 3  (7.1%) |
| Max HR (bpm) | 0  (0%) | 0  (0%) | 2  (4.8%) | 1  (2.4%) | 4  (9.5%) | 3  (7.1%) |
| ***Strength*** | | | | | | |
| Upper Body (reps) | 0  (0%) | 0  (0%) | 2  (4.8%) | 1  (2.4%) | 2  (4.8%) | 3  (7.1%) |
| Lower Body (reps) | 0  (0%) | 0  (0%) | 2  (4.8%) | 1  (2.4%) | 2  (4.8%) | 3  (7.1%) |
| ***Questionnaires*** | | | | | | |
| EQ-5D | 0  (0%) | 0  (0%) | 1  (2.4%) | 3  (7.1%) | 2  (4.8%) | 4  (9.5%) |
| FACT-P | 0  (0%) | 0  (0%) | 1  (2.4%) | 3  (7.1%) | 2  (4.8%) | 4  (9.5%) |
| BFI | 0  (0%) | 0  (0%) | 1  (2.4%) | 3  (7.1%) | 2  (4.8%) | 4  (9.5%) |
| Godin Leisure Time Exercise Questionnaire (modified) | 0  (0%) | 0  (0%) | 1  (2.4%) | 3  (7.1%) | 3  (7.1%) | 4  (9.5%) |
